# *In vitro* Dissolution Profile at Different Biological pH Conditions of Hydroxychloroquine Sulfate Tablets Is Available for the Treatment of COVID-19

**DOI:** 10.3389/fmolb.2020.613393

**Published:** 2021-01-14

**Authors:** Thirupathi Dongala, Naresh Kumar Katari, Santhosh Kumar Ettaboina, Anand Krishnan, Murtaza M. Tambuwala, Kamal Dua

**Affiliations:** ^1^Aurex Laboratories LLC, East Windsor, NJ, United States; ^2^Department of Chemistry, School of Science GITAM Deemed to be University, Hyderabad, India; ^3^Department of Chemical Pathology, Faculty of Health Sciences and National Health Laboratory Service, School of Pathology, University of the Free State, Bloemfontein, South Africa; ^4^School of Pharmacy and Pharmaceutical Science, Ulster University, Coleraine, United Kingdom; ^5^Discipline of Pharmacy, Graduate School of Health, University of Technology Sydney, Ultimo, NSW, Australia; ^6^Priority Research Centre for Healthy Lungs, Hunter Medical Research Institute (HMRI) & School of Biomedical Sciences and Pharmacy, The University of Newcastle (UoN), Callaghan, NSW, Australia; ^7^School of Pharmaceutical Sciences, Shoolini University of Biotechnology and Management Sciences, Solan, India

**Keywords:** coronavirus disease 2019, hydroxychloroquine sulfate, dissolution, validation, pH

## Abstract

Hydroxychloroquine sulfate is one of an extensive series of 4-aminoquinolines with antimalarial activity. Moreover, it is used for the treatment of rheumatoid arthritis. Sometimes, hydroxychloroquine sulfate is beneficial for the treatment of autoimmune diseases. Based on recent clinical experiments, it is exploited for the treatment of COVID-19, coronavirus across the globe. The chromatogram separation was achieved by using Agilent, Zorbax C8, 250 mm × 4.6 mm i.d., column. The buffer consists of 0.01 M of 1-pentane sulfonic acid and 0.02% of orthophosphoric acid in purified water. Mixed buffer, acetonitrile, and methanol (800:100:100 v/v). The flow rate was 1.0 ml min^−1^, and injection volume was 10 μl. Detection was made at 254 nm by using a dual absorbance detector (DAD). The reversed-phase high-performance liquid chromatography (RP-HPLC) method has been developed and validated as per the current International Conference on Harmonization (ICH) guidelines to estimate hydroxychloroquine sulfate tablets. As part of method validation, specificity, linearity, precision, and recovery parameters were verified. The concentration and area relationships were linear (*R*^2^ > 0.999) over the concentration range of 25–300 μg ml^−1^ for hydroxychloroquine (HCQ). The relative standard deviations for precision and intermediate precision were <1.5%. The proposed RP-HPLC generic method was applied successfully to evaluate the *in vitro* dissolution profile with different pH conditions such as 0.1 N HCl, pH 4.5 acetate buffer, and pH 6.8 phosphate buffers as US-marketed reference products.

## Introduction

Hydroxychloroquine (HCQ) is a white crystalline powder, chemically named 2-[4-[(7-chloroquinolin-4-yl)amino]pentyl-ethylamino]ethanol. It is easily soluble in water and organic solvents. HCQ is mainly used as a medication for malaria and a wide variety of inflammatory conditions. Moreover, HCQ has *in vitro* activity for the treatment of severe acute respiratory syndrome coronavirus 2 (SARS-CoV-2) and related coronaviruses problems (Colson et al., 2020; Wang et al., 2020; Yao et al., 2020). In recent days, it is proven to have the superficial ability and tolerable safety against coronavirus disease 2019 (COVID-19)-related pneumonia in clinical experiments performed in China (Gao et al., 2020) and other parts of the world. COVID-19, which appeared in the middle of December 2019, has proliferated swiftly, now confirmed in numerous countries. As of September 25, 2020, COVID-19 has produced 32,475,585 infections and 987, 754 deaths in all countries majorly in the USA, India, Brazil, Russia, and other countries across the globe.

**Figure 1:**
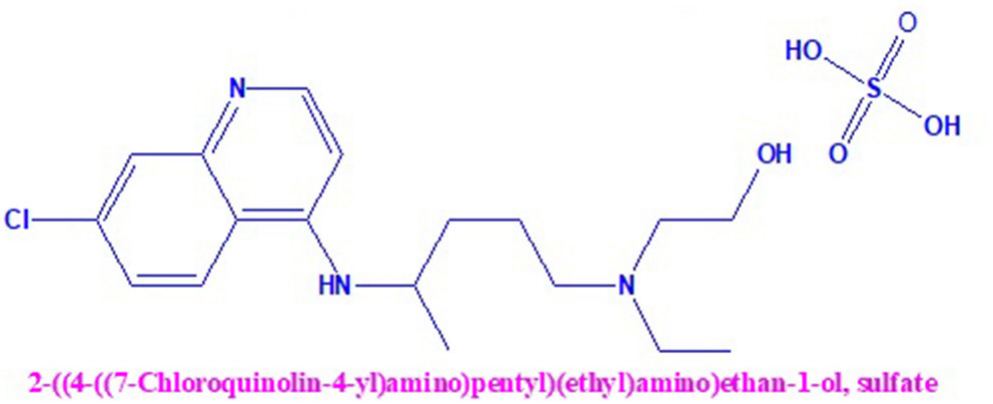
Chemical structure of Hydroxy chloroquine sulfate.

After conducting multiple clinical trials by the State Council of China, it confirmed that chloroquine phosphate, a long-standing medicine used for malaria treatment, had proven noticeable efficacy and satisfactory safety in treating COVID-19-related pneumonia patients. Serious unfavorable reactions to HCQ and chloroquine phosphate were not observed in the COVID-19 patients. Based on these conclusions, a conference was organized on February 15, 2020; members including professionals and experts from government and regulatory authorities of clinical trials agreed and approved that HCQ and chloroquine phosphate are effective against COVID-19. Based upon limited available clinical trial data, HCQ recommends the treatment of COVID-19-infected patients in most countries. Due to the wider accessibility of these HCQ tablets in the United States, it has been administered to hospitalized COVID-19 patients. It is still under clinical investigation trials to treat patients with different stages such as mild, moderate, and severe COVID-19. The chemical structure of HCQ is shown in Figure 1.

Currently, most countries were looking for HCQ drug product literature and other related information for better understanding due to the COVID 19 pandemic. It is essential to know the drug product release with respective time in *in vitro* conditions. The literature survey revealed that different types of analytical techniques were used for the determination of HCQ, such as high-performance liquid chromatography (HPLC) with photodiode array (PDA) detector (Volin, 1995; Zuluaga-Idárraga et al., 2014), HPLC with UV detector (Akintonwa et al., 1983; Morris, 1985; Brown et al., 1986; Tonnesen et al., 1988; Croes et al., 1994), chromatography-tandem mass spectrometry (MS/MS) (Wang et al., 2012; Füzéry et al., 2013), liquid chromatography (LC)/ion trap (IT)/MS (Dongre et al., 2009), capillary-LC (Chaulet et al., 1993), and electrochemical study (Arguelho et al., 2003).

Majority of the literature was reported in the pharmacokinetics of HCQ in biological fluids. The United States Pharmacopeia (USP) monograph method was reported with a UV-Spectro photometric technique for the estimation of HCQ tablets *in vitro* dissolution profile. Generally, the UV methods' results are not reliable due to the lack of reproducibility. Other than the USP method, there was no literature on HCQ tablet's *in vitro* dissolution profile in different multimedia to understand the product behavior. The reversed-phase (RP)-HPLC is a powerful and simple technique for quantification of drug products in different pH conditions.

Moreover, the regulated pharmaceutical industry released products into the global market after determining quality, safety, and efficacy. Hence, HPLC methods are required with suitable precision, accuracy, and sensitivity level. The present work's main aim is to develop a simple and reproducible HPLC method for estimation of HCQ dissolution profile (0.1 N HCl, pH 4.5 sodium acetate, pH 6.8 phosphate buffer).

## Materials and Methods

### Instrumentation

The Agilent HPLC 1260 Infinity-II was used for the estimation of HCQ dissolution profile. It consists of four channels, pressure range up to 600 bar, degasser with an integrated purge valve, thermostatic sampler, and column compartment. The PDA detector connected to empower 3 software (Build 3471 SPs Installed: Feature Release 3 DB ID: 2639633283) to monitor the output signal. The Disteck Premiere 5100 model dissolution apparatus was used to perform the multimedia profile. The column is Agilent Zorbax C8, 250 mm × 4.6 mm i.d., 5 μm. Sartorius analytical balances were used for the weighing of standards and samples. Bio-Technics ultra sonicator is used to extract the drug from the sample matrix.

### Materials and Reagents

The HCQ with certified purity of 99.2% was obtained from SCI pharma, Taiwan. AR grade ortho phosphoric acid, hydrochloric acid, potassium dihydrogen phosphate, 1-pentane sulfonic acid, NaOH, sodium acetate, and acetic acid chemicals were purchased from VWR chemicals, Radnor, PA, USA. HPLC-grade acetonitrile (99.9%) and methanol from J. T. Baker were procured from VWR chemicals, Radnor, PA, USA. High-quality HPLC-grade purified water was used throughout the experiments.

### Chromatographic and Dissolution Conditions

The chromatogram separation was achieved by using Agilent, Zorbax C8 (250 × 4.6 mm i.d.) 5.0 μm column with a simple isocratic method. The buffer consists of 0.01 M of 1-pentane sulfonic acid and 0.02% orthophosphoric acid in purified water. Mixed buffer, acetonitrile, and methanol (800:100:100 v/v). The flow rate was 1.0 ml min^−1^, and injection volume was 10 μl. Detection was made at 254 nm by using a dual absorbance detector (DAD). The dissolution conditions media volume 900 ml, USP-II (Paddle), and 50 rpm were used to perform the profile.

### Preparation of Dissolution Media Buffer

To prepare acidic medium, 8.5 ml of concetration HCl (35%) into 1,000 ml of volumetric flask and made up to volume with water. For pH 4.5 sodium acetate buffer, weighed and transferred 2.99 g of sodium acetate (NaC_2_H_3_O_2_.3H_2_O) into a 1,000-ml volumetric flask added 14 ml 2 N acetic acid (CH_3_COOH) and made up to volume with water. We prepared pH 6.8 phosphate buffer by mixing 250 ml of 0.2 M monobasic potassium phosphate solution and 112 ml of 0.2 M NaOH into 1,000 ml volumetric flask and made up to volume with water.

### Dissolution Conditions and Preparation of Sample

To evaluate the dissolution profile of HCQ reference listed product [Reference Listed Drug (RLD)] (Plaquenil) with USP apparatus-II (Paddle) having 50 rpm and 900 ml of medium maintained 37.0°C temperature. After dropping the RLD tablets into six individual dissolution vessels, we collected the samples at different time points 5, 10, 15, 20, 30, 45, and 60 min and injected into HPLC. The equal concentration of HCQ standard was prepared to calculate the percentage of drug release at each time point.

## Results and Discussion

### Optimization of Conditions

**Figure 2:**
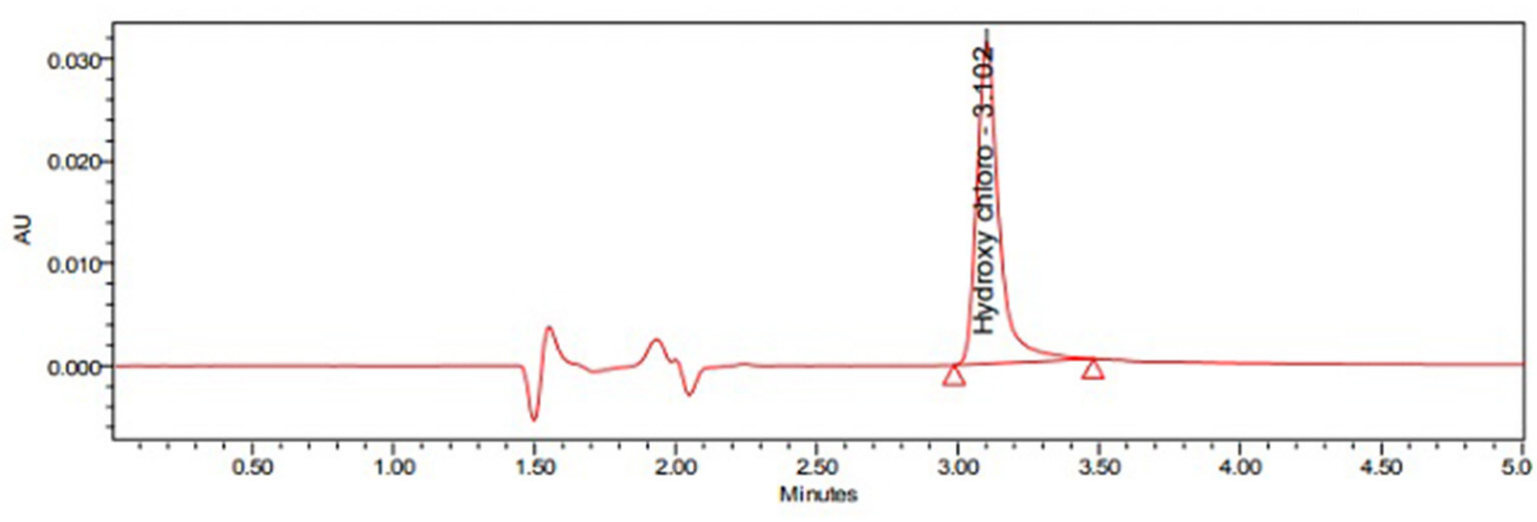
Typical Chromatogram of Hydroxy chloroquine sulfate standard.

The current study's main aim is to develop a simple and rapid HPLC method for the estimation of HCQ dissolution profile in different media. During the initial method, development scanned HCQ standard solution in PDA detector observed maximum UV absorption was observed at 254 nm; hence, this wavelength was fixed to optimize other chromatographic conditions. The major changes in the chromatographic conditions tested were explained in Table 1. The initial mobile phase compositions containing water and acetonitrile provided low HCQ interaction with the Zorbax column, resulting in rapid drug elution. Similarly, mobile phases containing a higher percentage of acetonitrile resulted in early HCQ elution (<1 min), showing peak with insufficient symmetry factors and plate count. HCQ is an easily water-soluble compound, which presents a pKa equal to 4–5. At pH below this value, HCQ molecule polarity is diminished, offering better drug interaction with C8 column. In this way, 1-pentane sulfonic acid and phosphoric acid solution were used as the buffered solvent, producing improved results. The buffer, methanol, and acetonitrile mixture at 800:100:100 (v/v) offered the most satisfactory HCQ peak separation with suitable retention time (3.1 min; Figure 2). The flow rate of HPLC was set to 1.0 ml min^−1^, and the injection volume was 10 μl. The evaluated system suitability parameters are retention time, tailing factor, and theoretical plates with optimized conditions (Table 2).

**Table 1:** Variation in analytical method conditions.

**Experiment Numbers**	**Mobile phase composition (Volume:Volume)**	**Injection volume (μl)**	**Flow rate (ml/min)**	**Results**
Trail-1	Acetonitrile and water (50:50)	50	1.0	Early elution and peak tailing
Trail-2	Acetonitrile and water (30:70)	50	1.0	Early elution and peak tailing
Trail-3	Methanol:Water (50:50)	50	1.2	Peak tailing and less plate count
Trail-4	Methanol:Water (20:80)	50	1.2	Peak tailing and broad peak
Trail-5	Acetonitrile:Methanol:Water (30:20:50)	30	0.8	Peak split and early elution
Trail-6	Acetonitrile:Methanol:Buffer (20:20:60)	30	1.0	Early elution and peak tailing
Trail-7	Acetonitrile:Methanol:Buffer (10:10:80)	10	1.0	Suitable peak

*HCQ samples (220.0 μg/ml) were analyzed in HPLC equipment using a C8 column, with UV detection fixed at 254 nm, at 30°C*.

*HCQ, hydroxychloroquine; HPLC, high-performance liquid chromatography*.

**Table 2:** System suitability results.

**S. No**.	**Name of the parameter**	**Observed value**
1	The USP Tailing factor of HCQ, peak from standard chromatogram	1.21
2	%RSD for peak area of HCQ for five replicate injections	0.24
3	The USP Theoretical plates of HCQ peak from standard chromatogram	5,568

*%RSD, % relative SD; HCQ, hydroxychloroquine; USP, United States Pharmacopeia*.

### Method Validation

The method validation was performed as per the current International Conference on Harmonization (ICH) guidelines. As part of method validation, specificity, linearity, precision, and recovery parameters were verified.

#### Specificity

Specificity is a significant parameter. Here, the optimized test method conditions are intended to estimate HCQ drug release from solid oral dosage formulations. We performed the specificity of the method by injecting the blank and placebo samples individually. We have evaluated the interference of unwanted peaks at the retention time of HCQ. The results show there was no interference at the retention time of the HCQ peak; hence, the optimized method is specific for the estimation of HCQ drug release in the dissolution profile.

#### Linearity

To perform the method's linearity, prepared a series of concentrations to range from 25 to 300 μg ml^−1^ of HCQ. The calibration curve was plotted for peak areas and concentration of HCQ standard solution. The concentration and area relationships were linear (*R*^2^ > 0.999) over the concentration range of 25–300 μg ml^−1^ for HCQ.

#### Precision

To evaluate the current optimized method's precision, prepared six sample solutions individually from the same pharmaceutical formulation were analyzed consecutively. The repeatability of the method was proven by calculating the six samples % relative standard deviation (%RSD). In the current method, the %RSD of six samples was found <2.0. In the similar way performed intermediate precision with different analysts, different days, and different dissolution apparatus, the %RSD of six samples was found <2.0.

#### Accuracy

The method's accuracy was carried out measuring the pharmaceutical samples fortified with a known quantity of the analytes. Spiked the known amount of HCQ at three different concentration levels (50, 100, and 150%) to placebo solution. Each concentration level was prepared in triplicate and calculated the recovery of each sample and RSD. The recovery results were between 97.0 to 103.0%, and RSD values were <2.0% (Table 3). The recovery values proved that the method allows direct determination of HCQ in tablet formulation in the presence of other excipients.

**Table 3:** Precision, intermediate precision (RSD), and accuracy of the HPLC method for HCQ quantification.

**Theoretical concentration (μg/ml)**	**Experimental concentration** **(mean ± SD;** **μg/ml)**	**RSD^a^ (%)**		**Accuracy^b^**
		**Precision**	**Intermediate precision**	
100.0	99.3 ± 1.15	1.16	1.05	99.3
200.0	200.8 ± 0.38	0.19	0.52	100.4
300.0	300.4 ± 0.85	0.28	0.63	100.1

a RSD = (SD/mean * 100);

b *accuracy = (experimental concentration/theoretical concentration) × 100*.

*HCQ, hydroxychloroquine; HPLC, high-performance liquid chromatography; RSD, relative SD*.

#### Solution Stability

The solution stability was performed at benchtop conditions. Prepared two samples as per the optimized method and kept on the benchtop for about 48 h. It was evaluated every 12 h with a freshly prepared standard solution. The HCQ sample solution was stable at benchtop for about 48 h. The samples were filtered through 0.45 μ nylon and polyvinylidene fluoride (PVDF) filters, there was no difference between the filtered and unfiltered samples.

#### Application of the Method

**Figure 3:**
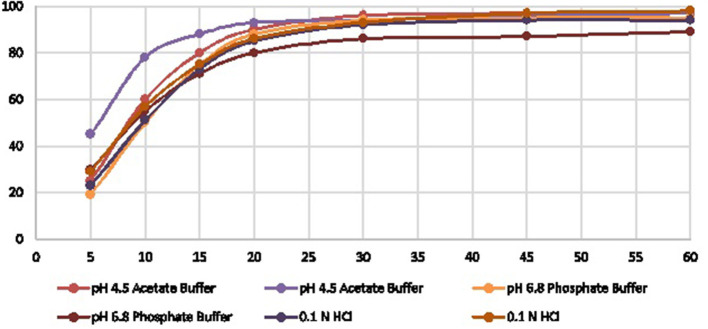
Graph model comparison of HCQ dissolution profile of Reference Vs In House.

The proposed RP-HPLC generic method was applied to analyze US-marketed products successfully and performed *in vitro* dissolution profile for the RLD and in-house formations to compare the product release with the respective specified time intervals. To complete the dissolution profile, we selected three different media such as 0.1 N HCl, pH 4.5 acetate buffer, pH 6.8 phosphate buffer to understand the product behaviors in low to high pH conditions. The results were shown different drug release pattern up to the first 15–20 min. After that, all the media reached optimum release (Table 4, Figure 3).

**Table 4:** Dissolution profile of HCQ RLD and in-house formulation results.

**Time**	**pH 4.5 Acetate buffer**				**pH 6.8 Phosphate buffer**				**0.1 N HCl**			
	**Reference**		**In-house**		**Reference**		**In-house**		**Reference**		**In-house**	
	**%**	**% RSD**	**%**	**% RSD**	**%**	**% RSD**	**%**	**% RSD**	**%**	**% RSD**	**%**	**% RSD**
**5 min**	25	36	45	18.4	19	37.6	30	16.4	23	23.9	29	14.1
**10 min**	60	21.3	78	8.8	50	17.3	55	16.6	51	17.2	57	16.5
**15 min**	80	13.8	88	6.8	75	12.1	71	15.8	73	14	75	13.1
**20 min**	90	7.7	93	4	88	5.9	80	13.8	85	10	86	8.7
**30 min**	96	3.2	94	3	94	2.6	86	11.5	92	3.6	93	3.9
**45 min**	97	2.1	96	2.4	95	1.6	87	10.1	94	1.2	97	1.8
**60 min**	97	1.6	97	2	95	1.6	89	9.1	94	1.2	98	1.2
**Recovery**	98	1.2	98	1.7	96	1.9	98	1.2	95	0.9	98	1.2

*HCQ, hydroxychloroquine; RLD, Reference Listed Drug; RSD, reference SD*.

## Conclusion

HCQ is currently recommended for the treatment of hospitalized COVID-19 patients in most of the countries in the world. It is essential to understand the drug profiling *in vitro* conditions; hence, a simple HPLC method has been developed successfully for estimation of the HCQ dissolution profile in *in vitro* conditions. The optimized HPLC method is used for the estimation of the dissolution profile of RLD and in-house tablets. The optimized method was validated as per the ICH guidelines, and the results were found satisfactory. Finally, the developed method was used in the quality control lab for the analysis of dissolution.

## Data Availability Statement

The original contributions presented in the study are included in the article/supplementary materials, further inquiries can be directed to the corresponding author.

## Author Contributions

TD and SE are the research scholars and did the literature and experimental work. NK has given the guidance to work and developed the draft. KA was monitored the research work. MT was helped *in vitro* dissolution studies at different biological pH conditions. KD was supported a lot to improve the quality of the manuscript. All authors contributed to the article and approved the submitted version.

## Conflict of Interest

TD and SE were employed by the company Aurex Laboratories LLC, 10 lake drive, East Windsor, NJ, USA 08512. The remaining authors declare that the research was conducted in the absence of any commercial or financial relationships that could be construed as a potential conflict of interest.
